# The mTORC2/AKT/VCP axis is associated with quality control of the stalled translation of poly(GR) dipeptide repeats in C9-ALS/FTD

**DOI:** 10.1016/j.jbc.2023.102995

**Published:** 2023-02-09

**Authors:** Yu Li, Ji Geng, Suman Rimal, Haochuan Wang, Xiangguo Liu, Bingwei Lu, Shuangxi Li

**Affiliations:** 1 Shandong Provincial Key Laboratory of Animal Cell and Developmental Biology, School of Life Sciences, Shandong University, Qingdao, China; 2 Department of Pathology, Stanford University School of Medicine, Stanford, California, USA

**Keywords:** C9-ALS/FTD, poly(GR), APP, TDP-43, FUS, mTORC2/AKT/VCP axis, AD, Alzheimer’s disease, APP, amyloid precursor protein, DPR, dipeptide repeat, FTD, frontotemporal dementia, FUS, fused in sarcoma, HEK, human embryonic kidney, MICOS, mitochondrial contact site and cristae organizing system, mTORC2, mechanistic target of rapamycin complex 2, OE, overexpression, RQC, ribosome-associated quality control, TDP-43, TAR DNA-binding protein 43, VCP, valosin-containing protein

## Abstract

Expansion of G4C2 hexanucleotide repeats in the chromosome 9 ORF 72 (*C9ORF72*) gene is the most common genetic cause of amyotrophic lateral sclerosis (ALS) with frontotemporal dementia (C9-ALS/FTD). Dipeptide repeats generated by unconventional translation, especially the R-containing poly(GR), have been implicated in C9-ALS/FTD pathogenesis. Mutations in other genes, including TAR DNA-binding protein 43 KD (TDP-43), fused in sarcoma (FUS), and valosin-containing protein, have also been linked to ALS/FTD, and upregulation of amyloid precursor protein (APP) is observed at the early stage of ALS and FTD. Fundamental questions remain as to the relationships between these ALS/FTD genes and whether they converge on similar cellular pathways. Here, using biochemical, cell biological, and genetic analyses in *Drosophila* disease models, patient-derived fibroblasts, and mammalian cell culture, we show that mechanistic target of rapamycin complex 2 (mTORC2)/AKT signaling is activated by APP, TDP-43, and FUS and that mTORC2/AKT and its downstream target valosin-containing protein mediate the effect of APP, TDP-43, and FUS on the quality control of C9-ALS/FTD–associated poly(GR) translation. We also find that poly(GR) expression results in reduction of global translation and that the coexpression of APP, TDP-43, and FUS results in further reduction of global translation, presumably through the GCN2/eIF2α-integrated stress response pathway. Together, our results implicate mTORC2/AKT signaling and GCN2/eIF2α-integrated stress response as common signaling pathways underlying ALS/FTD pathogenesis.

Amyotrophic lateral sclerosis (ALS) is a muscle wasting disease characterized by degeneration of lower motor neurons and axons and loss of upper motor neurons and their corticospinal tracts. Frontotemporal dementia (FTD) is a progressive neuronal atrophy with neuronal loss in the frontal and temporal cortices and associated behavioral and personality changes and impairment of language skills. Advances in human genetics have identified multiple genetic mutations commonly associated with ALS and FTD, revealing that these two diseases are related clinically, pathologically, and mechanistically and may represent a continuum of a broad neurodegenerative disorder (1, 2, 3).

Hexanucleotide expansion consists of GGGGCC (G4C2) repeats within the first intron of *C9ORF72* gene, which is the most common genetic cause of ALS/FTD (4, 5). Proposed mechanisms of C9-ALS/FTD pathogenesis include loss of normal C9ORF72 function, RNA-related toxicity due to RNA foci formation, and proteotoxicity associated with dipeptide repeats (DPRs) translated from expanded sense- and antisense *C9ORF72* transcripts (6, 7, 8, 9). Expanded *C9ORF72* mRNAs can be translated into five DPRs in a repeat-associated non-AUG translation mechanism, resulting in poly(GR), poly(GA), poly(GP), poly(PR), and poly(PA) formation (4, 5, 10, 11). All of the DPRs are found in brain tissues of ALS/FTD patients (12). Increasing evidence showed that the R-containing poly(GR) peptide exerts strong cellular toxicity *in vivo* in mice, *zebrafish*, *Drosophila*, and yeast models (13, 14, 15, 16, 17, 18, 19, 20, 21) and that abnormal poly(GR) protein aggregation and toxicity is tightly correlated with neurodegenerative phenotypes (22). Overexpression (OE) of poly(GR) leads to disruption of membrane-less nucleolar structures (7), global translation reduction (23), increased DNA damage (24), alteration of mitochondrial contact site and cristae organizing system (MICOS) morphology, disruption of ion homeostasis, and compromised mitochondrial function (17, 25).

Mutations in other genes that are commonly linked to ALS/FTD have also shed lights on disease pathogenesis. These include TAR DNA-binding protein 43 (TDP-43) (26) and fused in sarcoma (FUS) (27, 28), two DNA- and RNA-binding proteins with normal functions ranging from transcription and splicing to mRNA transport and translation, microRNA biogenesis, and stress granule formation (1). Other genes linked to ALS/FTD include valosin-containing protein (VCP), a member of the AAA ATPase family with established function in the recycling and degradation of ubiquitinated proteins, and genes with functions in protein clearance or maintenance of protein homeostasis, including ubiquilin 2 (UBQLN2), vesicle-associated membrane protein–associated protein B, p62/sequestosome 1 (SQSTM1), optineurin (OPTN), and charged multivesicular body protein 2B (CHMP2B) (1, 29). In addition, upregulation of amyloid precursor protein (APP), a protein whose aberrant processing or metabolism having been implicated in Alzheimer’s disease (AD), was observed at early stages of ALS and FTD, presumably as a compensatory response to neuronal damage or impairment of axonal transport (30). However, the relationships among the various ALS/FTD genes remain underexplored.

Given the prevalence of C9-ALS/FTD and the importance of poly(GR) to C9-ALS/FTD pathogenesis, it is imperative to understand cellular mechanisms underlying the quality control of poly(GR). Previous studies of protein quality control have focused on how proteins were handled after translation, *e.g.*, by chaperone-mediated refolding or proteasome- and lysosome-mediated degradation. However, recent studies reveal that problems with proteostasis are prevalent even with translating nascent peptide chains still associated with ribosomes, necessitating ribosome-associated quality control (RQC) mechanisms (31, 32). During translation elongation, ribosome slowdown and stalling can occur for various reasons. Some are functional and serve to facilitate cellular dynamics. Others are detrimental and can be triggered by damaged mRNAs, mRNA secondary structures, insufficient supply of aminoacyl-tRNAs or termination factors, or environmental stress (33, 34, 35, 36, 37, 38). In the case of poly(GR), some of which was translated on the surface of mitochondria and cotranslationally imported into the organelles (25), it was shown that its translation was frequently stalled, presumably due to positively charged arginine residues interacting with negatively charged residues lining the exit tunnel of 60S ribosome. Stalled poly(GR) translation activates the RQC process, the inadequacy of which can lead to the accumulation of aberrant, C-terminally modified (CAT-tailed) poly(GR) species that can perturb proteostasis and contribute to poly(GR) accumulation and neuromuscular degeneration (39).

In this study, we set out to test whether the other ALS/FTD-associated genes may participate in the quality control of poly(GR). Strikingly, we discovered that OE of APP, FUS, and TDP-43 restrains poly(GR) protein expression. Mechanistically, APP, FUS, and TDP-43 act through the mechanistic target of rapamycin complex 2 (mTORC2)/AKT/VCP axis to regulate the RQC of poly(GR) translation. Inhibition of the mTORC2/AKT/VCP axis could restore poly(GR) protein expression attenuated by APP, FUS, or TDP-43. Our data strongly implicate the mTORC2/AKT/VCP axis as a major regulator of protein quality control in ALS/FTD.

## Results

### APP OE significantly reduces poly(GR) protein expression

**Figure 1:**
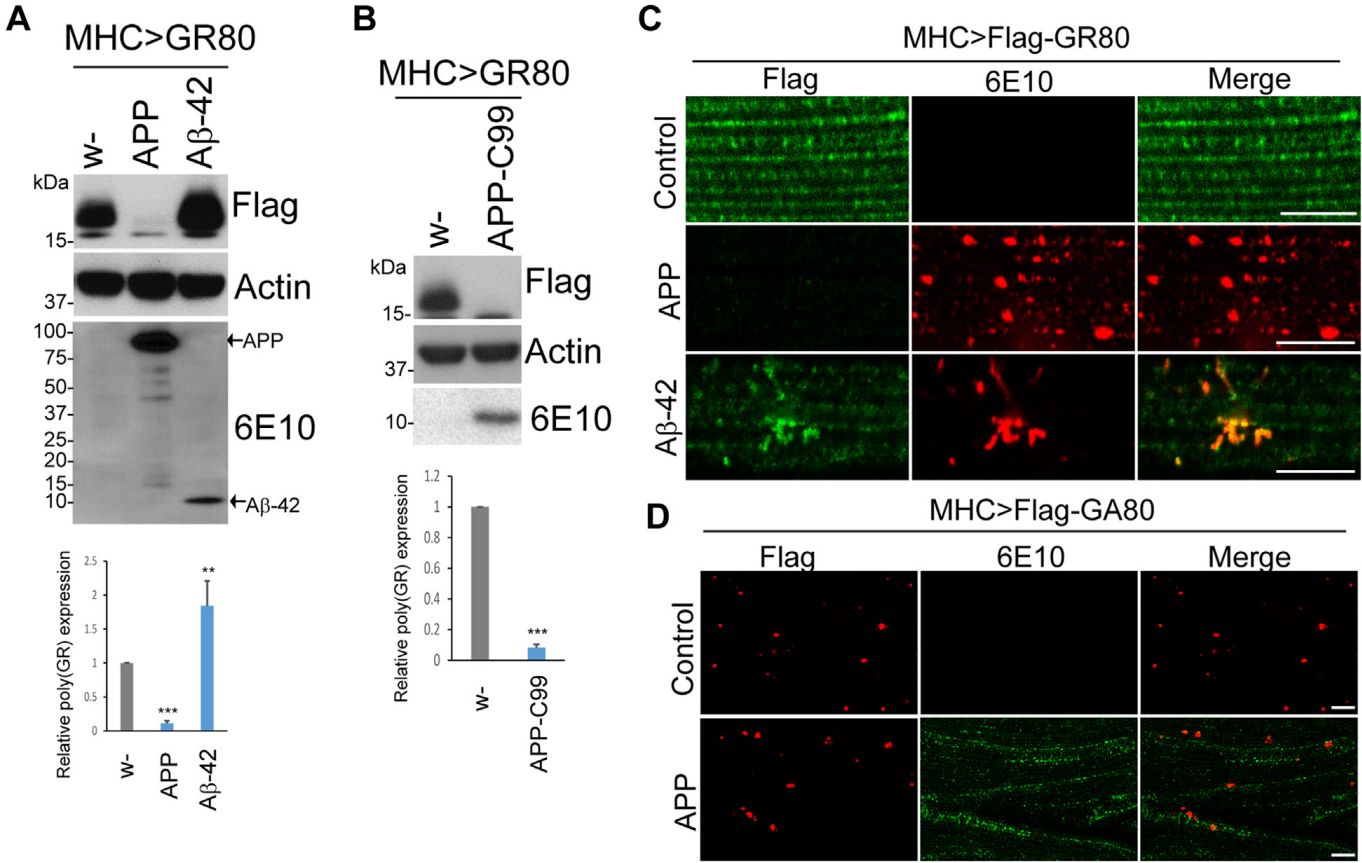
**APP overexpression restricts poly(GR) expression in *Drosophila* muscle.***A*, Western blot results showing Flag-(GR)80 expression in the presence of APP and Aβ-42. The 6E10 antibody is detecting APP and Aβ-42. Bar graph shows quantification of the relative expression of (GR)80. (∗∗∗*p* < 0.001; ∗∗*p* < 0.01) *B*, immunoblot results showing effect of APP-C99 coexpression on Flag-(GR)80 level in fly thoracic muscle extracts. Actin serves as loading control. Bar graph shows quantification of the relative expression of (GR)80 (∗∗∗*p* < 0.001). *C*, immunostaining of Flag-(GR)80 with Flag antibody and APP/Aβ-42 with 6E10 antibody in *Mhc>Flag-(GR)80* flies coexpressing APP or Aβ-42. The scale bars represent 8 μm. *D*, immunostaining of Flag-(GA)80 and APP with Flag and 6E10 antibody, respectively, in *Mhc>Flag-(GA)80* flies coexpressing APP. The scale bars represent 10 μm. APP, amyloid precursor protein.

APP is pivotal in the pathophysiology of AD, where APP is processed into β-amyloid peptides (Aβ40 and Aβ42) that are the main components of amyloid plaques in diseased brain (40). Expansion of G4C2 repeats in *C9orf72* has been found in clinical AD patients (41, 42). However, possible interplay between APP and C9orf72 repeat expansions has not been tested. We chose *Drosophila* as an *in vivo* system for this study, as it has been widely used for investigating ALS pathogenesis (13, 43, 44, 45, 46, 47, 48, 49, 50, 51, 52). In a *Drosophila* model expressing Flag-tagged GR80 repeats in the muscle using the *Mhc-Gal4* driver (*Mhc>Flag-GR80*) (25), we found that OE of APP robustly reduced GR80 expression level. In contrast, poly(GR) became more stable when Aβ-42 was coexpressed, suggesting that APP regulates poly(GR) protein level independent of the Aβ-42 region (Fig. 1*A*). To investigate the involvement of other APP fragments, we examined the effect of the C-terminal 99 amino acid fragment of APP-C99 on poly(GR) level. Consistently, OE of APP-C99 also led to diminished poly(GR) level (Fig. 1*B*). Since Aβ-42 corresponds to the first 42 amino acids of APP-C99, this result indicates that the 42 to 99 amino acid region of APP-C99 is involved in poly(GR) regulation. To further validate this result, we performed immunostaining of fly muscle tissue and observed that poly(GR) expression was indeed diminished upon APP OE (Fig. 1*C*). In contrast, Aβ-42 colocalized with poly(GR) and enhanced poly(GR) protein level (Fig. 1*C*). We next investigated the effect of APP on poly(GA), which was shown to be localized as inclusions in fly muscle (53). However, there was no change of poly(GA) level upon APP OE, supporting the specificity of poly(GR) regulation by APP (Fig. 1*D*).

### APP acts through the mTORC2/AKT/VCP axis to regulate poly(GR)

**Figure 2:**
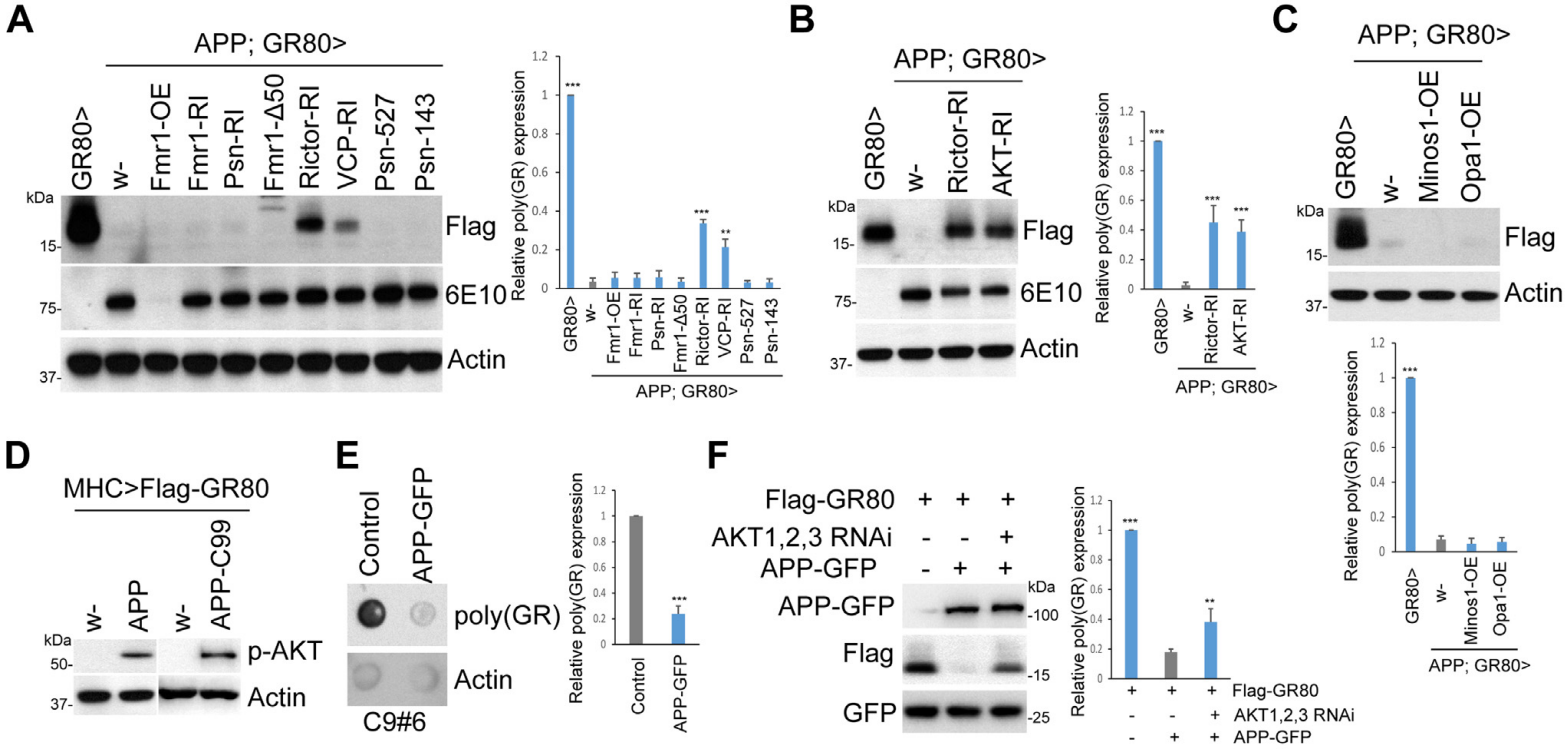
**APP regulates poly(GR) expression through mTORC2/AKT/VCP axis.***A*, WB analysis of Flag-(GR)80 protein expression levels in *Mhc>APP/Flag-(GR)80* flies after various genetic manipulations. Immunoblots are probed with the indicated antibodies. RI is abbreviation for RNAi and OE for overexpression. *B*, Western blot results showing that knockdown of Rictor or AKT can partially rescue Flag-(GR)80 expression in *Mhc>APP/Flag-(GR)80* flies. *C*, overexpression of MICOS components has no effect on Flag-(GR)80 expression in *Mhc>APP/Flag-(GR)80* flies. *D*, effect of APP or APP-C99 on AKT phosphorylation. *E*, dot blot analysis of endogenous GR level in C9ALS patient fibroblasts with or without APP-GFP transfection. *F*, effect of combined RNAi of AKT1, AKT2, AKT3 on Flag-GR80 expression in HEK293T cells with or without APP-GFP coexpression. Plasmid expressing GFP is used a transfection control. Bar graphs in panels represent quantification of the relative expression of (GR)80 (∗∗∗*p* < 0.001; ∗∗*p* < 0.01). ALS, amyotrophic lateral sclerosis; APP, amyloid precursor protein; HEK, human embryonic kidney; MICOS, mitochondrial contact site and cristae organizing system; mTORC2, mechanistic target of rapamycin complex 2; VCP, valosin-containing protein.

Next, we sought to understand the molecular mechanisms by which APP regulates poly(GR) expression. A genetic interaction approach was carried out to identify potential factors that could partially or fully restore the expression of GR80 protein attenuated by APP. As our previous studies identified the mTORC2/AKT/VCP signaling axis in regulating the RQC of poly(GR) translation (39), we focused on this pathway. We found that the knockdown of mTORC2 component Rictor, or VCP, a component of the RQC complex and a downstream effector of mTORC2/AKT signaling axis (39), were able to partially rescue poly(GR) expression. In contrast, the manipulation of Fmr1, a regulator of translation (54), and Presenilin 1, a component of the gamma-secretase involved in processing of APP and other transmembrane proteins (55), had no obvious effect (Fig. 2*A*). Furthermore, the knockdown of AKT efficiently restored GR80 expression attenuated by APP (Fig. 2*B*). Interestingly, although MICOS was previously identified as a mitochondrial structure that recruits poly(GR) and stabilizes it (25), it did not appear to mediate the effect of APP in poly(GR) regulation, as the OE of MICOS component Mic10 (Minos1) or Opa-1 failed to modulate the APP effect (Fig. 2*C*). Importantly, we found that AKT phosphorylation at the mTORC2 phosphorylation site was increased when APP or APP-C99 was overexpressed (Fig. 2*D*), demonstrating that APP acts upstream of mTORC2-AKT to regulate the RQC of poly(GR) translation.

To assess the disease relevance of our findings, we investigated the effect of APP on poly(GR) expression in C9-ALS/FTD patient fibroblasts. We found that APP OE lead to downregulation of poly(GR), supporting a conserved role of APP in controlling poly(GR) expression (Fig. 2*E*). We further checked whether the effect of APP on poly(GR) depended on AKT, which would be consistent with the observation in *Drosophila*. Mammals express three *AKT* genes. The combined knockdown of *AKT1*, *AKT2*, and *AKT3* in human embryonic kidney 293 (HEK293T) cells partially restored poly(GR) protein attenuated by APP OE (Fig. 2*F*). Together, our data support that APP acts upstream of the mTORC2/AKT axis to regulate the RQC of poly(GR) translation.

### FUS or TDP-43 OE restrains poly(GR) protein expression

**Figure 3:**
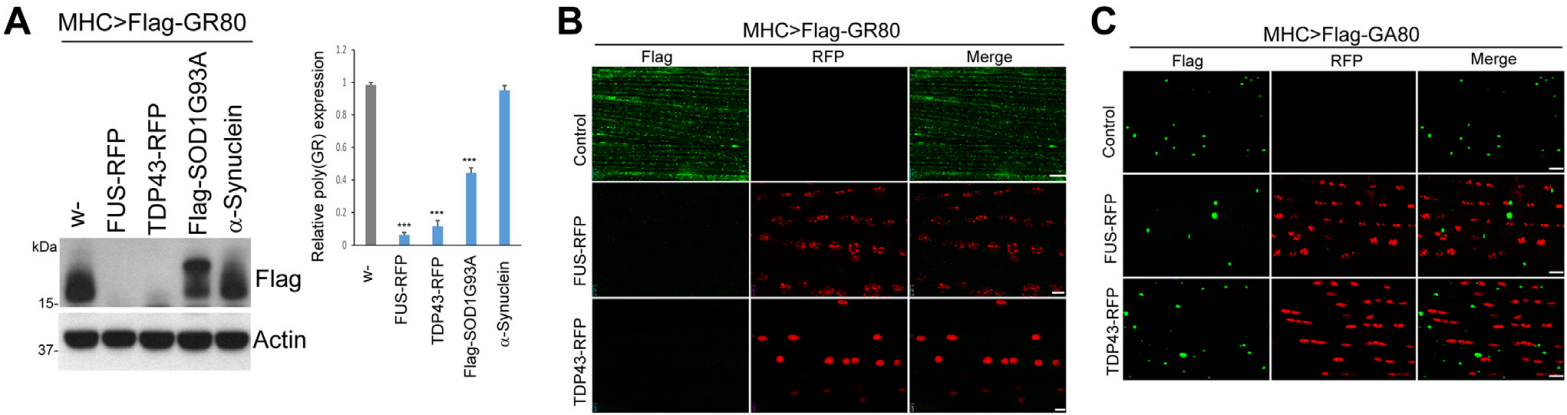
**FUS or TDP-43 coexpression diminish poly(GR) expression in *Drosophila* muscle.***A*, Western blot results showing effects of FUS, TDP-43, SOD1-G93A, or α-Synuclein coexpression on Flag-(GR)80 level in *Mhc>Flag-(GR)80* flies. The upper band in SOD1 lane represents Flag-SOD1-G93A. Bar graph shows quantification of the relative expression of (GR)80 (∗∗∗*p* < 0.001). *B*, immunostaining of Flag-(GR)80 and RFP in *Mhc>Flag-(GR)80* flies coexpressing RFP-tagged FUS and TDP-43. The scale bars represent 10 μm. *C*, immunostaining of Flag-(GA)80 and RFP in *Mhc>Flag-(GA)80* flies coexpressing RFP-tagged FUS and TDP-43. The scale bars represent 10 μm. FUS, fused in sarcoma; TDP-43, TAR DNA-binding protein 43.

We also investigated possible interplay between FUS or TDP-43 and poly(GR) using similar strategy as we did with APP. We found that OE of FUS or TDP-43 dramatically diminished poly(GR) expression (Fig. 3*A*). In contrast, OE of SOD1-G93A, which is associated with ALS but not FTD, or OE of α-synuclein, which is associated with familial Parkinson’s disease (56), did not affect poly(GR) expression (Fig. 3*A*), demonstrating the specificity of FUS and TDP-43 effects on poly(GR) expression. To further validate the FUS and TDP-43 effect on poly(GR), we performed immunostaining in fly muscle and found that poly(GR) protein was indeed dramatically inhibited in the presence of FUS or TDP-43 (Fig. 3*B*). The OE of FUS or TDP-43 had no obvious effect of GA80 expression in the muscle (Fig. 3*C*), demonstrating specificity of FUS and TDP-43 action in regulating poly(GR) expression during RQC. Thus, FUS and TDP-43 specifically restrain poly(GR) expression.

### The mTORC2/AKT/VCP axis mediates the effect of FUS and TDP-43 on poly(GR)

**Figure 4:**
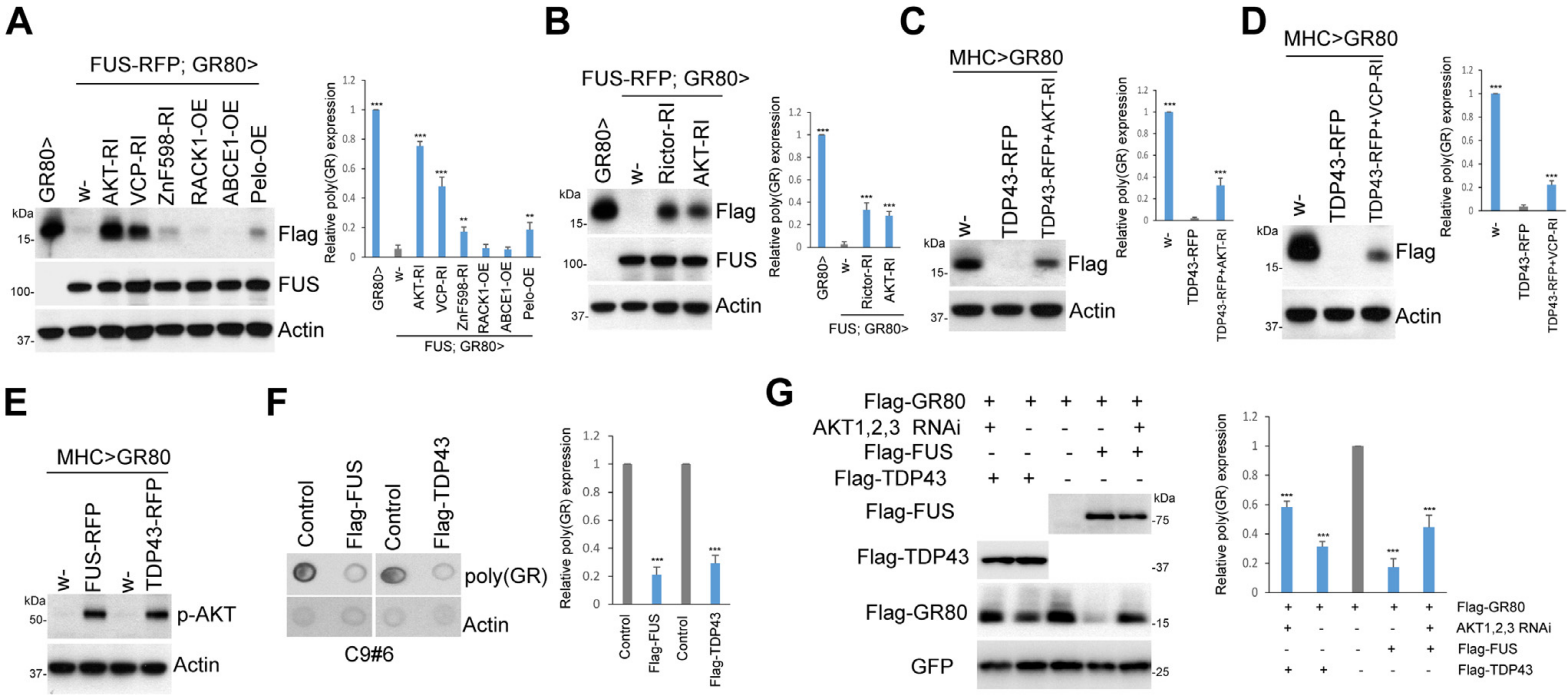
**FUS and TDP-43 modulate poly(GR) expression through the mTORC2/AKT/VCP axis.***A* and *B*, Western blot analysis showing effect of various genetic manipulations of RQC factors (*A*) or mTORC2/AKT pathway components (*B*) on the suppression of Flag-(GR)80 expression by FUS-RFP in *Mhc>Flag-GR80/FUS-RFP* flies. *C* and *D*, Western blot analysis showing effect of knockdown of AKT (*C*) or VCP (*D*) on the suppression of Flag-(GR)80 expression by TDP43-RFP in *Mhc>Flag-GR80/TDP43-RFP* flies. *E*, effect of FUS and TDP-43 on AKT phosphorylation level in *Mhc>Flag-(GR)80 flies*. *F*, dot blots showing effect of FUS and TDP-43 on poly(GR) expression in C9ALS patient fibroblasts. *G*, effect of combined RNAi of AKT1, AKT2, and AKT3 on Flag-GR80 expression in HEK293T cells with or without Flag-FUS or Flag-TDP43 coexpression. Plasmid expressing GFP is used a transfection control. Bar graphs show quantification of the relative expression of (GR)80 in all panels (∗∗∗*p* < 0.001; ∗∗*p* < 0.01). ALS, amyotrophic lateral sclerosis; FUS, fused in sarcoma; HEK, human embryonic kidney; mTORC2, mechanistic target of rapamycin complex 2; RQC, ribosome-associated quality control; TDP-43, TAR DNA-binding protein 43; VCP, valosin-containing protein.

As in the case of APP, we found that the knockdown of AKT, VCP (Fig. 4*A*), or Rictor (Fig. 4*B*) could partially restore poly(GR) protein expression attenuated by FUS, suggesting that the mTORC2/AKT/VCP axis plays an important role in mediating the effect of FUS on the RQC of poly(GR) translation. Similarly, the RNAi of VCP and AKT (VCP-RI and AKT-RI) could restore poly(GR) expression attenuated by TDP-43 OE (Fig. 4, *C* and *D*), supporting that the mTORC2/AKT/VCP axis also mediates the effect of TDP-43 on the RQC of poly(GR) translation. Next, we examined the phosphorylation status of AKT. The OE of FUS or TDP-43 significantly promoted AKT phosphorylation (Fig. 4*E*), suggesting that FUS and TDP-43 act upstream of mTORC2/AKT signaling to regulate the RQC of poly(GR) translation.

To further assess disease relevance, we examined possible interplay between FUS or TDP-43 and poly(GR) in C9-ALS/FTD patient fibroblasts. We found that FUS or TDP-43 OE led to a significant reduction of poly(GR) expression, supporting conserved roles of FUS and TDP-43 in regulating poly(GR) translation (Fig. 4*F*). To test whether the regulation of poly(GR) by FUS and TDP-43 was dependent on AKT signaling, we used HEK293T cells overexpressing Flag-GR80. We found that the simultaneous knockdown of all three AKT isoforms (AKT1, AKT2, AKT3) could partially restore poly(GR) protein attenuated by FUS or TDP-43, indicating that AKT is a key mediator of the effect of FUS and TDP-43 on poly(GR) translation (Fig. 4*G*).

### Global protein translation is repressed by poly(GR) and further exacerbated by APP, FUS, and TDP-43

**Figure 5:**
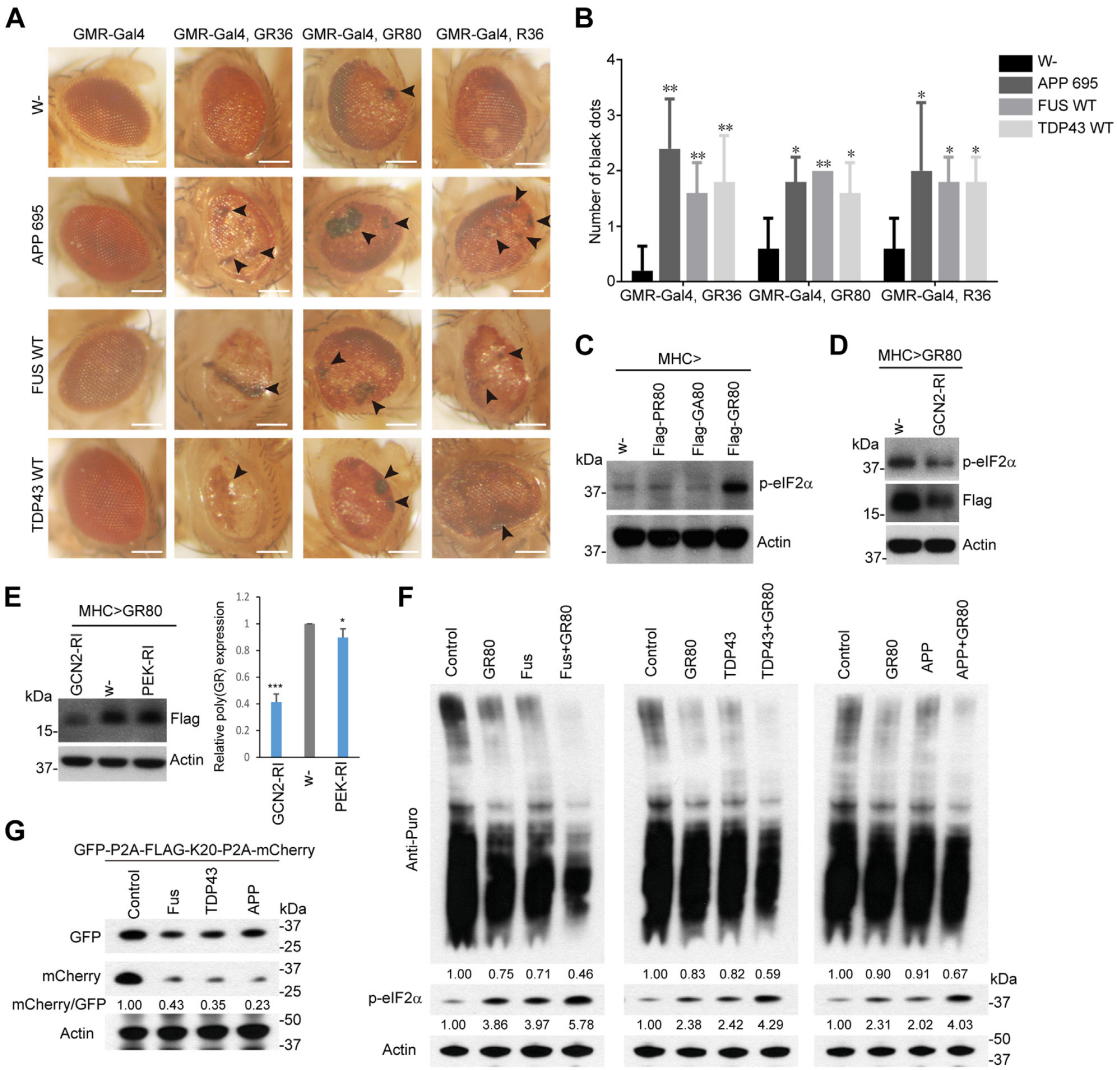
**General translation are inhibited when APP, FUS, and TDP-43 regulate poly(GR) in*Drosophila* diseases model. diseases model.***A*, representative bright-field microscope images of fly eye morphology in GMR-Gal4–driven GR36, GR80, or R36 transgenic flies, with or without the coexpression of APP, FUS-wt, or TDP-43-wt. *Arrowheads* mark necrotic spots. The scale bars represent 200 μm. *B*, graph quantifying the number of necrotic dots present on the eye surfaces shown in *A*. Values represent mean ± SD, ∗∗*p* < 0.01, ∗*p* < 0.05 vs *w*-control group. n = 5 for each genotype. *C*, Western blot analysis showing effect of the overexpression of GA80, PR80, and GR80 on phospho-eIF2α level. *D*, Western blot analysis showing effect of knockdown of the eIF2α kinase GCN2 on phospho-eIF2α level and Flag-GR80 level in *Mhc>Flag-(GR)80* flies. *E*, Western blot analysis showing effect of knockdown of GCN2 or PERK on Flag-GR80 level in *Mhc>Flag-(GR)80* flies. Bar graph shows quantification of the relative expression of (GR)80 (∗∗∗*p* < 0.001; ∗*p* < 0.05 ). *F*, Western blot analysis of the incorporation of puromycin into newly synthesized proteins in HEK293T cells transfected with GR80, FUS, TDP-43, APP, or cotransfected with GR80/FUS, GR80/TDP-43, and GR80/APP. *G*, Western blot analysis of the effect of APP, FUS, and TDP-43 on GFP and mCherry expression in HEK293T cells in the GFP-P2A-FLAG-K20-P2A-mCherry translational reporter assay. The mCherry/GFP ratio reflects extent of translation stalling, with lower ratio meaning stronger stalling and *vice versa*. APP, amyloid precursor protein; FUS, fused in sarcoma; HEK, human embryonic kidney; TDP-43, TAR DNA-binding protein 43.

We sought to determine the functional consequence of the APP, FUS, and TDP-43 regulation of the RQC of poly(GR) translation. Although many of the *in vivo* poly(GR) protein expression studies were carried out in fly muscle, the OE of APP, FUS, and TDP-43 alone in the fly muscle is toxic, resulting in abnormal wing posture phenotypes similar as what was caused by GR80 (25), making it difficult to perform genetic interaction studies. We next used the fly eye as an experimental system to examine possible genetic interactions. OE of APP, FUS, or TDP-43 in the fly eye using GMR-Gal4–driven expression of the *UAS* transgenes we tested did not have obvious effect on eye morphology, making it possible to perform genetic interaction studies. We used three models of C9ALS-FTD: GR36, GR80, and R36, expressing 36 copies of GR, 80 copies of GR, or 36 copies of G4C2 repeats, respectively, for the genetic interaction studies. In the case of GR36 (13), its expression driven by GMR-Gal4 driver resulted in small and depigmented eyes. Coexpression of APP, FUS, or TDP-43 resulted in male lethality. In the few females that eclosed, their eyes were collapsed, smaller, and contained more necrotic black spots than in *GMR-Gal4>GR36* alone (Fig. 5, *A* and *B*). In the case of GR80, GMR-Gal4–driven expression is lethal, therefore the conditional expression system was used to generate viable *GMR-Gal4>UAS-GR80; tub-Gal80*^*ts*^ flies (57), which exhibited rough eyes with occasional necrotic black spots. Coexpression of APP, FUS, or TDP-43 significantly increased the number of necrotic black spots (Fig. 5, *A* and *B*). Similar result was observed with the *GMR-Gal4>R36* model (13), although in this case the *GMR-Gal4>R36* eye morphology was more regular than the other models, but coexpression of APP, FUS, or TDP-43 significantly increased the number of necrotic black spots on the eye surface (Fig. 5, *A* and *B*).

Our observation of APP, FUS, or TDP-43 OE reducing GR80 expression in multiple systems, yet enhancing the toxicity of GR36, GR80, and R36 in the fly eye was paradoxical. To try to understand the mechanism involved, we hypothesized that induction of stalled ribosomes during poly(GR) translation may generate a stress signal that recruits APP/FUS/TDP-43 and the downstream mTORC2/AKT/VCP pathway for ribosome-mediated quality control. However, prolonged activation of this pathway may lead to problems with global translation. Supporting this notion, phosphorylation of eIF2α, a central marker of integrated stress response (58), was increased in flies expressing poly(GR) but not poly(GA) or poly(PR) (Fig. 5*C*). Knockdown of the eIF2α kinase GCN2 reduced p-eIF2α level induced by GR80 (Fig. 5*D*), supporting the involvement of GCN2 in the integrated stress response in the *Drosophila* poly(GR) model. Interestingly, knockdown of GCN2, but not another eIF2α kinase PERK, reduced poly(GR) expression as well (Fig. 5, *D* and *E*).

One possibility is that reducing p-eIF2α by loss of GCN2 increased translational initiation, leading to more trailing ribosomes colliding with stalled GR80 ribosomes, eventually leading to further reduction of GR80 protein expression. Using puromycin labeling of nascent peptide chains to measure global translation in mammalian cells, we found that poly(GR) expression resulted in reduced global translation and that the coexpression of APP, FUS, and TDP-43 resulted in further reduction of global translation (Fig. 5*F*). The reduction of global translation was correlated with the increase of p-eIF2α level by poly(GR) and individual expression of APP, FUS, or TDP-43 and the further increase of p-eIF2α level when poly(GR) was coexpressed with APP, FUS, or TDP-43 (Fig. 5*F*), consistent with induction of the integrated stress response. To further test the effect of APP, FUS, and TDP-43 on stalled translation, we used a translational stalling reporter GFP-P2A-FLAG-K20-P2A-mCherry, in which K20 was used to induce ribosome stalling, the GFP was used to measure global translation, and mCherry/GFP ratio was used to measure ribosome readthrough of the K20 stall (59). We found that mCherry/GFP ratio were dramatically decreased when APP, FUS, or TDP-43 was coexpressed with the stalling reporter in HEK293T cells (Fig. 5*G*), indicating that APP, FUS, and TDP-43 attenuated readthrough of stalled translation. Moreover, the reduction of GFP expression by APP, FUS, or TDP-43 (Fig. 5*G*) suggested that they repressed the overall translation of stalled mRNAs.

**Figure 6:**
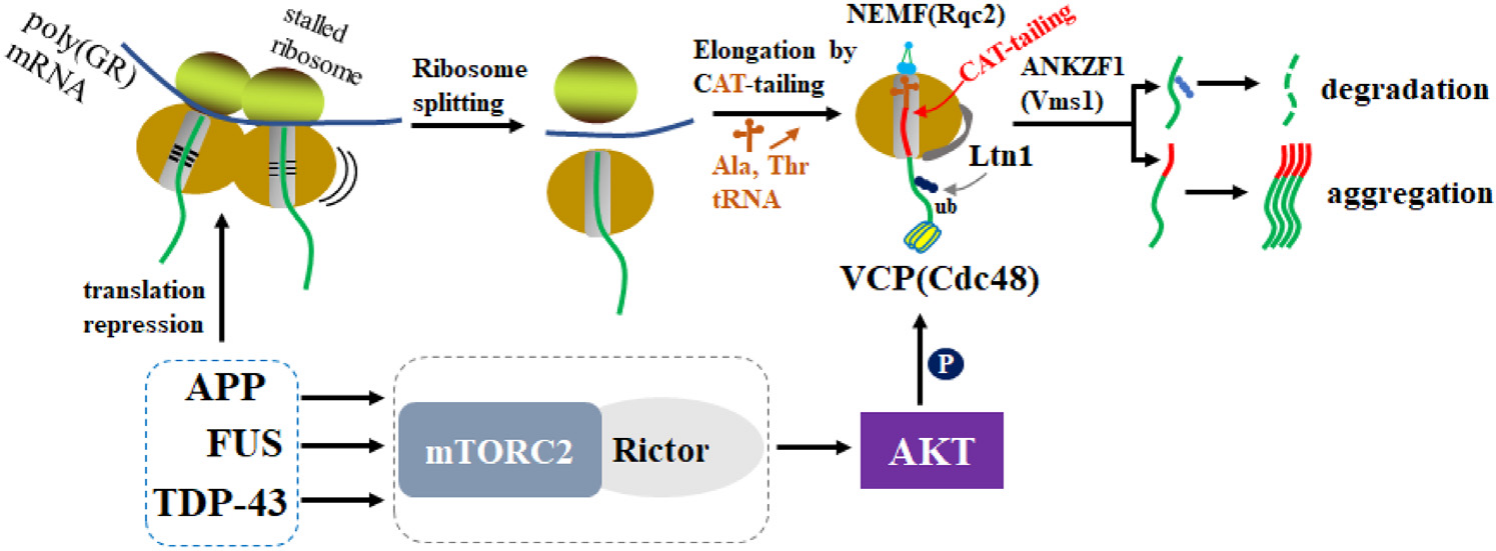
**Molecular mechanisms of how APP, FUS, and TDP-43 effects on poly(GR) expression.** A model illustrating the mechanism by which APP, FUS, or TDP-43 suppresses poly(GR) expression. One the one hand, APP/TDP43/FUS act as upstream regulators of the mTORC2/AKT/VCP axis to regulate the RQC of poly(GR). On the other hand, APP/TDP43/FUS represses the overall translation of stalled *poly(GR)* mRNA and possibly other stalled mRNAs. APP, amyloid precursor protein; FUS, fused in sarcoma; mTORC2, mechanistic target of rapamycin complex 2; RQC, ribosome-associated quality control; TDP-43, TAR DNA-binding protein 43; VCP, valosin-containing protein.

In summary, our data support the working model that APP, FUS, and TDP-43 are upstream regulators of the mTORC2/AKT/VCP axis, which regulates the RQC of poly(GR) during its translation stalling (Fig. 6). Moreover, our data suggest that APP, FUS, and TDP-43 can also induce the repression of global translation when ribosome stalling is persistent.

## Discussion

Rapid developments in human genetics studies have led to the discovery of a large number of genes associated with ALS/FTD (29). Deciphering the normal and pathological functions of these genes and their relationships in the disease process is an important prerequisite to a mechanistic understanding of the pathogenesis and future therapeutic development targeting ALS/FTD. Our genetic studies, with validation in mammalian cell culture system including C9-ALS/FTD patient fibroblasts, uncovered the mTORC2/AKT signaling axis regulating the RQC of C9-ALS/FTD-associated poly(GR) translation as a common cellular pathway involved in ALS/FTD. Our findings of the lack of effect of SOD1-G93A or α-Syn on poly(GR) and the lack of effect of FUS or TDP-43 on poly(GA), the translation of which may not be associated with ribosome stalling (39), strongly support the specificity of the genetic relationships among FUS, TDP-43, and C9-ALS/FTD-associated poly(GR) identified in this study.

Going beyond the genes genetically linked to ALS/FTD, we show that APP also acts through the mTORC2/AKT signaling axis to regulate the RQC of C9-ALS/FTD-associated poly(GR) translation. The involvement of APP in ALS has previously been studied in the context of ALS, and APP or its metabolite was found to exacerbate ALS-related phenotypes in the SOD1-G93A mouse model (60, 61). Our results suggest that APP can activate mTORC2/AKT signaling to alleviate stalled translation of poly(GR) and restrain the expression of aberrant poly(GR) translation products, at least at the initial stage. It is possible that in ALS/FTD setting, APP is upregulated as a protective response in response to neuronal damage at an early stage of disease as previously suggested (62), presumably caused by stalled translation and collided ribosomes. However, chronic upregulation of APP may contribute to disease due to the accumulation of APP metabolites, the stalled translation of APP itself (53), or the prolonged activation of stress response pathways by APP may lead to the depression of global translation. Consistent with this notion, we found that integrated stress response as indicated by eIF2α phosphorylation was heightened in transgenic flies expressing poly(GR). This is presumably caused by the ribosome stalling occurring during poly(GR) translation, as stalled ribosomes have been shown to activate the integrated stress response and other stress response pathways that can influence cell fates (63, 64, 65).

A similar situation may occur with TDP-43 and FUS. In fact, both TDP-43 (66) and FUS (67) have been shown to associate with stalled ribosomes, and in the case of TDP-43, its association with stalled ribosomes provides neuroprotection function in the face of sublethal stress (66). Intriguingly, we showed that the APP-C99 portion of APP is sufficient to activate the mTORC2/AKT axis and regulate GR80 translation, whereas the Aβ-42 portion of APP was without effect. This finding resonates with recent revelation of aberrant APP-C99 as the etiological driver of AD (53). Remarkably, the translation of APP-C99 is also frequently stalled, the inadequate RQC of which can generate aberrant translation products that precipitate hallmarks of AD (53). It is therefore fascinating that OE of one stalled translation product (APP-C99) would abrogate the stalled translation of another (GR-80). Future studies will investigate at the biochemical level on how APP/APP-C99, FUS, and TDP-43 signal to the mTORC2/AKT/VCP axis to regulate the RQC of stalled poly(GR) translation, whether endogenous stalled peptides that serve as RQC substrate(s) may also targeted by this pathway, and how this signaling process may be targeted for therapeutic purposes.

## Experimental procedures

### Fly genetics

Following fly strains were purchased from Bloomington, FlyORF, and VDRC *Drosophila* stock center. APP (BL#6700), APP-C99 (BL#33783), Aβ-42 (BL#32038), AKT-RI (BL#33615), AKT-RI (BL#82957), Rictor-RI (BL#36699), VCP-RI (V#24354), VCP-RI (BL#32869), ZnF598-RI (BL#61288), RACK1-OE (FlyORF# F001043), ABCE1-OE (FlyORF# F001097), Pelo-OE (BL#68150), Minos1-OE (FlyORF# F002914), UAS-Fmr1 (BL#6931), Fmr1-RI (BL#27484), Psn-RI (BL#38374), Psn-527 (BL#8306), Psn-143 (BL#8296), GCN2-RI (BL#67215), PEK-RI (BL#42499). TDP43-RFP and FUS-RFP were obtained from Dr Jane Wu (Northwestern U); UAS-Flag-GR80, UAS-Flag-GA80, and UAS-Flag-PR80 were shared by Dr Fen-biao Gao (U Mass); Opa1-OE fly stocks were from Dr Leo Pallanck. Flies were raised at 25 °C incubator on a standard food containing Water, 17 L; Agar, 93 g; Cornmeal, 1716 g; Brewer’s yeast extract, 310 g; Sucrose, 517 g; Dextrose, 1033 g. *Drosophila* genetic crosses were performed with standard procedures under a 12 h light/dark cycle.

### Western blot analyses

Fly muscle were homogenized in the lysis buffer [50 mM Tris–HCl, pH7.4, 150 mM NaCl, 5 mM EDTA, 10% glycerol, 1% Triton X-100, Protease inhibitor (cat#: B14012, Bimake)]. Similarly, cultured cells were lysed in the same buffer. After centrifugation at 10,000*g* for 5 min, the supernatant was subjected to Western blot analysis on NuPAGE 4% to 12% Bis–Tris Gels (cat# NP0321, Invitrogen) with different antibodies and transferred to polyvinylidene difluoride membrane with 0.2 μm pore size (Millipore). For dot blot assay, cell extracts were blotted on HybondTM-C super NC membrane, air-dried, blocked in 5% dry milk in TBST (PBS with 0.1% Tween-20), and incubated with indicated primary antibodies overnight at 4 °C. Antibodies for blotting and their dilutions were as follows: mouse anti-Flag (1:1000, Sigma-Aldrich, F1804), rabbit anti-Flag (1:1000, Sigma-Aldrich, F7425), mouse anti-actin (1:5000, Sigma, A2228), rabbit anti-Fus (1:1000, Proteintech, 11570-1-AP), rabbit phospho-AKT S473 (1:1000, Cell signaling, 9217), mouse anti-GFP (1:3000, Proteintech, 66002-1-Ig), β-amyloid (clone 6E10) (1:500, Biolegend, SIG-39320), poly-GR (1:500, Proteintech, 23978-1-AP), mouse anti-puromycin(1:1000, MABE343, Sigma), p-eIF2a(1:1000, Cell Signaling, 3597), and Rabbit anti-mcherry (1:1000, Proteintech, 26765-1-AP). Polyvinylidene difluoride and NC membranes were then incubated with secondary antibodies: Goat anti-Mouse IgG-HRP (Santa Cruz, sc-2005) and Goat anti-Rabbit IgG HRP (Santa Cruz, sc-2004) before processing with Konica Minolta SRX-101A medical film processor.

### Fly muscle immunoblotting

Dissected muscle samples were fixed with 4% formaldehyde for 30 min at room temperature. After washing with PBST (1× PBS with 0.2% Triton X-100) for 15 min each time, samples were subsequently blocked with 1× PBS containing 5% Bovine serum albumin followed by incubation with primary antibodies at 4 °C overnight. After washing with PBST for 15 min each time, samples were incubated with Alexa Fluor 594-conjugated and Alexa Fluor 488-conjugated (1:500, Invitrogen) for 2 h at room temperature and subsequently mounted in Slow-Fade mounting medium (Invitrogen). Antibodies used in the study were as follows: mouse anti-Flag (1:1000; Sigma) and β-amyloid (clone 6E10) (1:500, Biolegend, SIG-39320). For *Drosophila* muscle staining, eight individuals were examined for each genotype and the representative images were showed.

### Mammalian cell culture and transfection

C9-ALS patient fibroblasts were kindly provided by Dr Aaron Gitler (Stanford University). HEK293T cells (ATCC) and C9-ALS patient fibroblast were maintained under 37 °C with 5% CO_2_ in Dulbecco’s modified Eagle’s medium–high glucose (Sigma Aldrich) supplied with 10% fetal bovine serum. Transfections were performed according to manufacturer’s instructions. Plasmids transfections were carried out by using Lipofectamine 3000 (cat#: L3000015, Invitrogen), and siRNA knockdown experiments were performed using Lipofectamine RNAi-MAX reagent (cat#: 13778150, Invitrogen). pCDNA3-Flag-GR80 was described before (39). After 72 h transfection, cells were washed with 1× PBS and then subjected to lysis and Western blot analysis. siRNAs used in the study were purchased from Invitrogen: siCon (cat#: 12935-400), siAKT1 (VHSS40082), siAKT2 (VHS41339), and siAKT3(cat#: AM51331).

### Puromycin labeling of ribosome stalled newly synthesized proteins

HEK293T cells were transfected with Flag-GR80, TDP43-tdTomato (Addgene #28205), Flag-FUS (Addgene #44985), APP-GFP (Addgene #69924) plasmids using Lipofectamine 3000 following standard protocol. Forty eight hours later, cells were treated with puromycin (10 μg/ml) for 15 min to label newly synthesized proteins before harvesting. Cell lysates were prepared and subjected to Western blot analysis.

### Translational stalling reporter assay

HEK293T cells were transfected with K20 (GFP-P2A-FLAG-K20-P2A-mCherry) reporter together with TDP43-tdTomato, Flag-FUS, and APP-GFP for 48 h. Cells were collected and prepared for Western blot assay. Translation stalling was analyzed by calculation of mCherry *versus* GFP ratio.

### Statistical analysis

For the quantification of Western blot results, relative signal intensity was measured and calculated by using NIH Image J (https://imagej.nih.gov/nih-image/). Student’s *t* test and one-way ANOVA test were used for statistical evaluation. All data are represented as mean ± SD, ∗ stands for *p* < 0.05, ∗∗ stands for *p* < 0.01, ∗∗∗ stands for *p* < 0.001.

## Data availability

All data that support the findings of this study are available on the request from the corresponding author.

## Conflict of interest

The authors declare that they have no conflicts of interest with the contents of this article.
